# After you: conversations between patients and healthcare professionals in planning for end of life care

**DOI:** 10.1186/1472-684X-11-15

**Published:** 2012-09-17

**Authors:** Kathryn Almack, Karen Cox, Nima Moghaddam, Kristian Pollock, Jane Seymour

**Affiliations:** 1 School of Nursing, Midwifery and Physiotherapy, University of Nottingham, Queen’s Medical Centre campus, B Floor, South Block Link, Nottingham, NG7 2UH, UK; 2 Clinical Psychology, Bridge House 1207, University of Lincoln, Brayford Pool, Lincoln, LN6 7TS, UK

**Keywords:** Advance care planning, Palliative care services, Preferred place of care, Qualitative research

## Abstract

**Background:**

This study explores with patients, carers and health care professionals if, when and how Advance Care Planning conversations about patients’ preferences for place of care (and death) were facilitated and documented.

**Methods:**

The study adopted an exploratory case study design using qualitative interviews, across five services delivering palliative care to cancer and non-cancer patients within an urban and rural English region. The study recruited 18 cases made up of patients (N = 18; 10 men; 8 women; median age 75); nominated relatives (N = 11; 7 women; 4 men; median age 65) and healthcare professionals (N = 15) caring for the patient. Data collection included: 18 initial interviews (nine separate interviews with patients and 9 joint interviews with patients and relatives) and follow up interviews in 6 cases (involving a total of 5 patients and 5 relatives) within one year of the first interview. Five group interviews were conducted with 15 healthcare professionals; 8 of whom also participated in follow up interviews to review their involvement with patients in our study.

**Results:**

Patients demonstrated varying degrees of reticence, evasion or reluctance to initiate any conversations about end of life care preferences. Most assumed that staff would initiate such conversations, while staff were often hesitant to do so. Staff-identified barriers included the perceived risks of taking away hope and issues of timing. Staff were often guided by cues from the patient or by intuition about when to initiate these discussions.

**Conclusions:**

This study provides insights into the complexities surrounding the initiation of Advance Care Planning involving conversations about end of life care preferences with patients who are identified as having palliative care needs, in particular in relation to the risks inherent in the process of having conversations where mortality must be acknowledged. Future research is needed to examine how to develop interventions to help initiate conversations to develop person centred plans to manage the end of life.

## Background

There is some evidence that, where implemented, Advance Care Planning (ACP) has positive outcomes in terms of patients dying in their preferred place of care and death, increased satisfaction of family carers and reduced costs for health care [1-3]. This evidence often stems from research studies where ACP has been a study intervention with a particular focus on one aspect of ACP (preferred place of death). Evidence suggests that in usual practice, ACP discussions are uncommon and rarely documented [4,5]. Some research has investigated views of patients about ACP [6-9]. Other studies have addressed the challenges of ACP for different groups of Health Care Professionals (HCPs) such as general practitioners (GPs), community nurses (CNs) [10-13] and out of hours GPs [14]. This literature identifies issues about the timing, initiation, conduct and recording of discussions, communication and exchange of information between professionals. Overall, however, evidence about the communication practices necessary to enable engagement in ACP is limited. In particular, there has been little research exploring the perspectives of all parties concerned (HCPs, patients and family carers). This paper seeks to address this gap.

The paper reports on one element of a broader study which set out to investigate issues of choice and decision making in end of life care (EOLC) from the perspective of patients, their family members and HCPs involved in their care. One of the key objectives in our study related to the *Preferred Place of Care* (PPC^a^) tool. This originated as part of a District Nurse education programme [15-17] to encourage discussion of ACP. The study aimed to explore if, when and how PPC was used to facilitate conversations about patients’ preferences (for place of care and death) and how these were documented. Discussion and recording of these preferences is seen as an important means of supporting and enabling patient choice, currently a central aspect of EOLC policy in England [18]. PPC is one of three interventions that were rolled out in England in the first phase of the National End of Life Care Programme between 2004–2007. In addition, the Gold Standards Framework was developed as a grass roots initiative to improve palliative care within primary care settings^b^. The Liverpool Care Pathway is an integrated care pathway used at the bedside to deliver sustained quality of care for the dying in the last hours and days of life^c^.

The End of Life Care Strategy (EOLCS) for England [19] was published in 2008. This further emphasised the government’s core commitment to making excellent EOLC universally available through the realisation of patient choice about the manner and, particularly, the place of dying. It sets out an EOLC Pathway, the first step of which highlights that discussions about, and recording of, preferences for future care between people approaching the end of life, their family members and health and social care staff are central to the delivery of good EOLC. ACP was highlighted as a key area within the Strategy and it has subsequently become more clearly defined in policy and guidance^d^[19,20]. ACP has been defined as a ‘voluntary process of discussion and review to help an individual who has capacity to anticipate how their condition may affect them in the future and, if they wish, set on record: choices about their care and treatment and / or an advance decision to refuse a treatment in specific circumstances, so that these can be referred to by those responsible for their care or treatment (whether professional staff or family carers) in the event that they lose capacity to decide once their illness progresses’ [20]. The EOLC Strategy has identified the lack of open communication between people approaching the end of life, their family members and health and social care staff as one of the key barriers to the delivery of good EOLC. Poor communication about EOLC is a common and enduring complaint [21]. The point at which ACP discussions are initiated (when, by whom, with whom) is the critical juncture upon which all else hangs, and yet probably because this is a difficult issue to research, there has been little evidence about this crucial aspect of practice. This paper explores the factors influencing if, when and how ACP takes place between HCPs, patients and family members from the perspectives of all parties involved and how such preferences are discussed and are recorded.

## Methods

The study utilised a retrospective audit of care delivered in the last four weeks of life (this is reported on elsewhere [22]) which was followed by interviews with patients, their family carers and nominated HCPs about their experiences of palliative care provision including the initiation of conversations about patients’ preferred place of care and death. This element of the study was exploratory and pragmatic in nature with a focus on interactions between HCPs, patients and their families. In consultation with an advisory group, five care services (see Table 1) with involvement in palliative care were selected across one region, chosen to cover palliative care provision for cancer and non-cancer populations across organisational boundaries.

**Table 1:** Study sites

**Site**	**Type**	**Area of practice**
**1**	GP practice service using Gold Standards Framework (primarily cancer-focused)	The practice covers an urban area as well as the surrounding more sparsely populated rural areas. A team of district nurses is attached to the practice.
**2**	Heart failure community matron service (home based nursing service for people with heart failure living in the community)	This service covers a predominantly rural area. Approximately 40% of patients referred to the service die within a year of referral.
**3**	Hospital Specialist Palliative Care Service (primarily cancer- focused)	This team support the major acute hospital in one of the network trusts.
**4**	Non-GSF Nursing Care home (providing general palliative care)	This was a residential nursing care home (30 beds) in a rural area. At the time of our study, the care home had not adopted the GSFCH (Gold Standards Framework in Care Homes) but was using the Liverpool Care Pathway.
**5**	Hospital Heart Failure Clinic	This hospital based team provides information and support. Most referrals came from hospital cardiologists/nurses.

HCPs from each of the selected services were invited to take part in our study to participate in an initial group interview. From each service these HCPs were also asked to assist with recruitment of patients to the study. We asked HCPs to identify patients from their palliative care register^e^ using the “surprise” question (“would I be surprised if this patient died in the next year?”). This has been recognized as one means of improving EOLC by identifying patients with a poor prognosis [23]. HCPs had copies of the study’s information sheet to give to patients who they identified as potential study participants. If patients then expressed an interest in taking part in our study they were asked to contact the researchers listed on the information sheet or they gave their permission for HCPs to pass on their contact details for the researchers to make contact. Once patients had consented to be in the study and prior to the first interview, we asked the referring HCP to brief us on patients’ level of awareness about their condition and palliative care services; levels of awareness, as reported by the HCPs, varied. Once recruited, patients were asked to nominate a family carer/relative to be interviewed and a HCP involved in their care at home^f^ (quite often this was the same HCP who had referred them to our study). Informed consent was sought and gained from all participants. Tables 2 and 3 provide details on patient, relative and healthcare professional recruitment and data collected. Table 4 provides demographics for the sample of patients.

**Table 2:** Patient/relative recruitment

**Site**	**No. cases to participate in initial interview**	**No. cases to participate in follow up Interview**	**Additional Notes**
**1**	5 (5 patients; 4 relatives)	4 (3 patients; 3 relatives)	One follow up interview was conducted with 2 relatives of a patient who died soon after 1^st^ interview. One other patient died before follow up interview was arranged.
**2**	3 (3 patients; 4 relatives)	2 (2 patients, 2 relatives)	One patient died before follow up interview was arranged.
**3**	4 (4 patients; 1 relative)	0	Two patients died shortly after 1^st^ interview.
**4**	5 (5 patients; 2 relatives)	0	Although all 5 patients were still living at the end of the study, delay in access to this site precluded the possibility of follow up interviews
**5**	1 (1 patient; 0 relatives)	0	One patient died soon after 1^st^ interview.
**Total**	**18 cases (18 patients; 11 relatives)**	**6 cases (5 patients; 5 relatives)**	

Follow up interviews were conducted for 6 of the 18 cases up to one year after the first interview (2 separate interviews with patients; 3 joint interviews with patients and relatives; 1 interview with 2 relatives of a patient who had died since the first interview). There was a level of attrition that impacted on the number of follow up interviews we were able to conduct. In total 6 patients recruited died before a follow up interview was planned. A further 4 patients were known to have died very soon after the completion of fieldwork.

Recruitment from sites 3, 4 and 5 was delayed, leaving no time for follow up interviews.

In total we recruited:

· 9 patients with cancer (sites 1 and 4) - 7 died during or shortly after the study.

· 4 patients with heart failure (sites 2 and 5) - 3 died during or shortly after the study.

· 2 patients with multiple sclerosis (site 4).

· 3 patients who had had strokes and co-morbidities associated with old age (site 4).

(No patients from site 4 died during or in the period shortly after the completion of fieldwork).

**Table 3:** Health care professional recruitment

**Site**	**First group interview – numbers and composition**	**Follow up interview with patient nominated health care professional**
**1**	4	2 (joint interview)
	GP, District Nurse (2) Practice Manager	District Nurse (2)
**2**	2	2 (joint interview)
	Community Matron (2)	Community Matron (2)
**3**	3	2 (separate interviews)
	Macmillan nurse (2), Manager	Macmillan nurse (2)
**4**	4	1
	Manager, care co-ordinator, registered nurse (2)	Manager
**5**	2	1
	Heart failure nurse, Community Nurse Specialist	Community Nurse Specialist
**Total**	**15**	**8**

In total we recruited 15 staff to the initial group interviews.

Towards the end of the study we carried out interviews with 8 HCPs across the five sites. These HCPs were nominated by patients and all had also taken part in the group interviews.

These follow up interviews focused on exploring their views regarding the specific patient/family cases that we interviewed (with patients’ permission) and their experiences of the research process.

**Table 4:** Patient sample demographics

**Patient**	**Age**	**Sex**	**Diagnosis**	**Family circumstances**
P101	80	M	Prostate cancer	Lives with wife. Daughter and son live locally
P102	70	M	Kidney and metastatic cancer	Lives with wife. Son nearby
P103	33	F	Skin and metastatic cancer	Lives with husband and three children
P104	59	F	Lung cancer	Lives alone. Daughter and son live locally and support from her Church community
P105	83	F	Lung cancer	Lives with daughter and son-in-law
P201	79	M	Heart failure	Lives with wife. Three daughters, one son all live locally.
P202	61	F	Congenital heart failure	Lives with husband. Support from daughter though she does not live nearby.
P203	72	M	Heart failure	Lives with wife. Two daughters who live locally
P301	65	F	Breast and metastatic cancer	Lives with husband. Daughter lives locally
P302	77	M	Kidney and metastatic cancer	Lived alone. Since diagnosis has moved in with son and daughter-in-law. One daughter lives locally.
P303	73	M	Prostate cancer	Lives with wife. Two daughters, one son live locally
P304	69	M	Prostate cancer	Lives with wife. Son and daughter live locally
P401	90	F	Stroke	Family visit but live some distance away
P402	67	M	MS	Wife visits plus has day visits home. Two sons visit
P403	88	M	MS	Wife visits. Two sons visit.
P404	82	F	Stroke, Spondylosis	Son visits. One daughter who visits irregularly
P405	81	F	Stroke	No family
P501	88	M	Heart failure	Lives alone.

Participants from Site 4 (P401 to 405) lived in a care home.

In the initial interviews with patients, we asked them to tell us about their understanding of their illness and current state of health/illness. We explored how they felt about the care and support they were receiving from family, friends and HCPs and in their view, how well-informed they felt they had been by HCPs. We covered similar topics with relatives (sometimes in separate interviews and sometime in a joint interview with the patient – we discuss issues relating to this in a later section). Most relevant to this paper we explored experiences of discussions between patients, family, friends and with HCPs, including discussions about future care, preferences for where patients would like to be cared for and any documentation of those discussions. Similar themes were explored in the follow up interviews with an emphasis on exploring what may have changed or stayed the same and why, in terms of patient preferences. Towards the end of the study we invited HCPs involved in the study to take part in a follow up interview to reflect and comment on the individual clinical cases that they had referred to us (patient consent included agreement for us to discuss their case with a nominated healthcare professional). These follow up interviews provided an additional perspective on if and how discussions about PPC were initiated.

All interviews were digitally recorded then fully transcribed. Detailed analysis of the interview material was undertaken using a constant comparative technique [24]. The research team (all authors) initially read through a selection of interviews separately to identify identified themes emerging and then compared notes. This thematic analysis continued through regular research team meetings, readings and discussion of further interview transcripts and a coding framework was developed that KA and NM applied to a selection of transcripts. Codes applied were subsequently rationalised into one 56 item coding frame that was applied to all transcripts, assisted by NVivo software. Ethical approval was obtained from the Local Research Ethics Committee (ref no. 06/Q2404/123) and relevant NHS Trust approvals were also secured.

## Results

### Issues relating to the initiation of discussions around PPC

This section reports primarily on findings from patient and carer interviews, but with some commentary from the follow up interviews carried out with HCPs, which adds an additional perspective to the accounts provided by patients and family carers.

Of the 18 patients interviewed, 13 were cancer or heart failure patients. Of these 13, 9 had a degree of ‘open awareness’ [25]. They reported that they had engaged in some level of conversation with both family carers and/or HCPs about EOLC, although the depth, process and areas reported to have been addressed in these conversations varied. We employ the term ‘open awareness’ here to refer to patients’ acknowledgement of ‘certain death but at an unknown time’ [26]. The degree of ‘open awareness’ among the remaining four cancer and heart failure patients was harder to establish. Two (one cancer, one heart failure) explicitly stated they had no recall of having had any discussions with HCPs or family. However, in response to a question about preferences for future care they did outline their preferences for place of care and death in the interview (albeit briefly). A further two cancer patients reported having had no conversations with HCPs or family carers about their preferences for future care. They also closed off this question in the interview, as we discuss below.

The five participants who were in the nursing care home appeared least likely to have any degree of ‘open awareness’ or to have had conversations about their preferences for EOLC^g^. They were all long term residents (having lived in the care home between two and seven years); three (average age 84) had had strokes and two (average age 77) had MS. Two residents talked about their desire to return home to live (although in both cases care home staff and family members indicated that this was not a realistic option). In a follow up interview, the care home manager indicated that initiating conversations about residents’ preferences for EOLC was rarely a priority, particularly when somebody was first admitted (unless ‘admitted as a terminal individual’).

We specifically asked whether patients had a PPC document. Only two patients had PPC documents in place that they were able to locate and show to the researcher; two patients were uncertain as to whether they may have completed a PPC document; one patient knew that her preferences were recorded in her notes but had no PPC document. Thirteen patients did not have a PPC document nor any recall of preferences being documented elsewhere. We did not ask direct questions about issues such as ‘advance decisions to refuse treatment’, often known colloquially as ‘living wills’, and ‘lasting powers of attorney’ but in asking questions about planning for future care, these topics were conspicuous in their absence in interviews with both patients and HCPs^h^.

Four participants appeared not to have engaged in any significant communication about EOLC preferences with either family members or HCPs. A key factor appeared to be that at the time of interview these patients reported being at a stage where they didn’t want to think too far ahead^i^: For example, when asked if HCPs had initiated any conversations about her future care, one cancer patient responded:

No, not at this time because I don’t see myself as being that far down the road yet, I’m still quite positive, well apart from when I’m feeling really ill (P103, first interview).

This respondent also acknowledged:

… at the end of the day we know it’s serious … it’s not going to have a good ending but I just think that you’ve got to carry on fighting … (P103, first interview).

At the time of interview she had surpassed all expectations on her prognosis. The metaphor of fighting can be one way of coping. While HCPs understandably would not wish to detract from that strategy, potentially it precludes the possibilities of conversations looking further ahead and planning for EOLC.

It is also possible that patients had had such conversations but either did not recall these when asked in the interview or perhaps did not want, at that point in time, to revisit those conversations. In an interview with the nominated HCPs providing P103 with care, they reported raising the topic of future care when first involved in her case (not mentioned by the patient in her interview) but had subsequently found it very difficult to know how or whether to broach the topic again.

Other participants reported some initial conversations about future plans but indicated that these had not been revisited for some time. One patient with heart failure reported some conversations with HCPs during a period when he was seriously ill and required hospitalisation but he had not subsequently followed up on these conversations:

P203: I’ve been feeling pretty good now for about two or three months I suppose.

IV: So do those sorts of issues about (future plans) - do they go to the back of your mind when you’re feeling a bit better?

P203: Oh yeah, I don’t give them a thought …

(P203 – first interview)

This patient reported that when these conversations were initiated by HCPs, he wondered if they did so because he was close to dying; this may explain in part why he and the HCPs involved in his care had not revisited the discussions since, or had been reluctant to do so when he was feeling relatively better.

Another cancer patient reported not having had any conversations with HCPs about preferences for where he wanted to be cared for. However, in the interview he revealed that he had given some thought to future plans about where he wanted to be cared for and die.

IV: Has anybody talked to you about where you want to be cared for? In terms of staying at home or, has anyone had those sort of conversations with you?

P101: No, no, not yet. No. I certainly want to stay at home. I’ll be quite frank with you. If I’m going to die, I want to die at home; I don’t want to die in hospital. And the family, I think, understand that.

In a follow up interview with the nominated HCPs involved in the care of this patient (after his death), they recalled difficulties in knowing how and when to initiate conversations with him about his preferences:

He never really, up until the very end, particularly considered himself to be palliative. Only near the end did he say ’I don’t think I’m winning this’ and that was the first indication I had that he was thinking along the lines of ’I’m going to die from this’. (HCP. S1 FU).

This patient died suddenly from a heart attack. It can be very difficult for HCPs to judge timing of initiating conversations with patients. As identified by the HCPs in this case, that may mean that discussions about preferences are never raised:

… we never actually asked him where he would like to die. It was always a case of let’s see what’s happening with you and he steered you away from that all the time (HCP2, S1 FU).

This example illustrates some the complexities involved. The patient gave some indication of his preferences to the researcher about his wishes. However, the HCPs felt he steered them away from such conversations, such that it was perceived to be too difficult and possibly unethical to open up discussions about his preferences for EOLC.

Patients and relatives rarely gave lengthy accounts of discussions about preferences for place of care and death^j^. Only one set of interviews (joint initial and follow up interviews with a patient with cancer and his wife) provided a more extended illustration of ongoing conversations with HCPs on EOLC preferences. This patient had a PPC document in place, indicating a preference to be cared for and to die at home. At the follow up interview 6 months later the couple reported on discussions with the district nurses to address some of the practicalities as to how their preferences might be achieved, although the review and additional information was not recorded in their PPC document.

The nominated HCPs involved in the care of this patient reported that in this case it had been the patient and his wife had *initiated* conversations about EOLC and preferences:

HCP1: The first time I met them, I walked in and it was just as though it was a case of, it was like an open book

HCP2: We’ve talked to them about where he wants to die and what the future possibly holds and how she is going to cope, what services are available, that’s been a conversation we’ve had right from the beginning. It was initiated by them right at the beginning and a couple of times they’ve initiated it to re-visit (S1 FU).

It is interesting to note that the only case of apparently detailed discussion was initiated by the patient. The HCPs identified two key factors which enabled them to have initial and on-going conversations with the patient and his wife. First, that the couple were very open to having such conversations and second, that over time they developed a depth of rapport with this couple.

### Health care professionals’ reports of discussions with patients about preferences for end of life care

The above section presents HCPs views on specific cases, drawing on data from the follow up interviews carried out. Here we report briefly on more general reflections from the discussion group interviews with HCPs on a range of factors that influence if, when and how they initiate discussions about preferences for EOLC, summarized in Table 5.

**Table 5:** Raising the topic of PPC with patients: factors identified by healthcare professionals

Factors that influence IF conversations are initiated:	1. Barrier of inexperience: the need for training and developing experience in advanced communication skills
	2. Judgement call on patient’s level of awareness/denial
	3. Unwillingness of relatives to have these conversations
	4. Uncertainty of trajectory with long term conditions (heart failure)
Factors that influence WHEN conversations about PPC take place	1. Patients initiate or ask for information
	2. Judgement on timing – don’t want to concern patients/relatives too early (nor leave it too late)
	3. Once preparatory work is carried out (getting to know the patient; planning what to say)
	4. Because of pressure to follow policy guidelines and find out patient preferences
Factors that influence HOW these conversations take place	1. Taking a ‘drip drip’ approach
	2. Use of trigger questions
	3. Different choice of language e.g. some HCPs will use the words death and dying; some would not

Gauging patients’ level of awareness and/or denial (which may also be present at variable levels at different points in time) presents a key challenge for HCPs working with both heart failure and cancer patients. It can be difficult to have conversations about EOLC with patients who do not consider themselves to be in need of palliative care and/or who are seeking to maintain a positive attitude:

… if you think they’re coming towards end of life, with all the uncertainty around heart failure, you want to discuss that, but at the same time, you don’t want to take away all their hope (HCP2. S2 DGP)

A number of HCPs reported that they waited for patients or family carers to raise the issues themselves:

It’s very much led by the patient; if they want to know … how they are doing whatever, and be guided intuitively by them really. There are some patients who will be very open and frank with you and use all the right words but there are others that will say to you or indicate ’I know where you’re going with this and I don’t want to hear’ (HCP1. S3 DGP).

Thus, to some extent, HCPs tended to rely on patients to explicitly raise issues for discussion rather than initiate these themselves. At the same time they were alert to cues from the patient or guided by intuition as to when to introduce issues around EOLC, what depth to go into and so on.

Factors mentioned in interviews with HCPs regarding judgments on timing included doing preparatory work and first building up a relationship with the patient and family:

It’s important we’ve built up a rapport with the patient … and that’s why we like early referrals so we get to know the person (HCP1. S1 DGP).

Despite a preference for early referrals which enabled them to develop a relationship with the patient prior to raising sensitive and difficult issues, HCPs reported that a significant number of referrals are made ‘late’ i.e. in a patient’s last few weeks or days of life.

## Discussion

This study provides insights into the different perspectives of patients, family carers and HCPs relating to discussions about patients’ preferences for place of care and death. The findings indicate that this is a complex and sensitive area for all concerned. Our focus was on the PPC (Preferred Place of Care) tool, which at the time of our study was one of the main tools for good practice in EOLC in the UK (since renamed Preferred Priorities for Care). This remains the case but the policy framework around EOLC has also placed an increasing emphasis on ACP as a means of opening discussion relating to wider range of issues to be considered about care at the end of life.

However, on the ground, ACP is still a difficult topic to broach. Guidance on ACP for HCPs [27] acknowledges that there is no ‘right time’ to introduce the topic, although it is also suggested that it is important to open up communication to discuss preferences at the earliest opportunity. Our findings have relevance here in adding to knowledge of a range of factors that contribute to the potential for reticence, evasion, reluctance by all parties involved to broach conversations to discuss ACP and EOLC needs. Not all patients wanted to discuss preferences (for place of care and/or death) with their family and/or HCPs or within an interview setting, an ambivalence that is also identified in other studies [4,10,28,29]. In our study, it appeared that conversations about these issues occurred more frequently between patients and their family carers than with HCPs but in all cases, reports of ‘one off’ discussions were more common than descriptions of a process, revisited and reviewed over time, which elsewhere is recognized to be important [30]. Overall, in the interview setting, patients did not elaborate on conversations about preferences for EOLC. This reticence contrasted with the lengthier accounts given about diagnosis in which participants included detailed accounts of how this was delivered and their reactions to the news. Not only did patients and their family carers have little to say about PPC, but when asked, there was also little indication of any expressed needs to engage in such discussions, and this was the case across the study sites. While some patients expressed expectations that this would be a topic that HCPs would initiate, there was no sense from the data that patients and family carers felt dissatisfied where this had not been the case. There may be a number of reasons for patient and family carers’ reluctance to engage with this topic. Some did not see the need to have discussions or felt it was something for ‘further down the line’. It may have been that some lacked knowledge and/or awareness of the options and possibilities to discuss plans for future care. Similar findings were identified in a study of patients’ with pancreatic cancer in relation to discussions about ‘place of death’ [30]. Copp and Field [26] discuss how denial and acceptance of dying can fluctuate during the period of dying; these ‘strategies’ can form coping strategies and may also be employed within research interviews to protect oneself or others taking part in the interview.

Our findings indicated equal reticence on the part of the HCPs, who were often hesitant to take a lead for several reasons. These included concerns about causing distress, taking away hope or touching on topics that the patient was not ready to engage with. A key barrier for HCPs initiating conversations on the subject is a perceived concern about taking away any hope. However, there is some evidence to suggest that engaging in ACP discussions can positively enhance rather than diminish patients’ hopes [31,32].

Timing is another key issue identified in our study. The uncertain trajectory of patients’ ill health can present an additional difficulty for HCPs in judging when to introduce discussions about EOLC, particularly for patients with long term conditions [10,13,33,34]. HCPs frequently made judgement calls, often guided by intuition, on patients’ – and family carers’ – levels of awareness or denial. Indeed, the ambivalence of HCPs appears to have been influenced in part by their awareness and sensitivity to their patients’ receptivity to engage in discussions about aspects of ACP.

Several studies have reported that most patients find it acceptable to engage in ACP discussions at the time they are diagnosed with a long term condition and may wonder why such discussions are not raised by HCPs at this point in time [6,9,30]. This questioning of why HCPs had not done so did not feature in our study. HCPs may be making sensitive appropriate judgments calls, following patient cues. However, another study highlights the risk of taking such a cautious and indirect approach, and that this may in turn lead to inaccurate assumptions about patient preferences [34]. These issues warrant further investigation.

### Strengths and limitations of the study

Patients were referred to the study via HCPs who were asked to select individuals from their palliative care register using the “surprise” question (“would I be surprised if this patient died in the next year?”). However, the nature, relevance and ground for referrals to palliative care are not well-defined. It was difficult to ascertain the number and nature of interactions that patients had had with HCPs or the range of HCPs involved in this aspect of care. We had conversations with the referring HCP prior to the initial interviews with patients and their family members, seeking some information about patients’ degree of awareness about their condition and prognosis. We used this information to guide us to some extent in how far we explored patients’ perspectives on future care. Interviews were undertaken by researchers skilled in dealing with sensitive issues. However, establishing the degree of patients’ ‘open awareness’ was not always easy; we aimed to ask the same questions but were more tentative in our approach to probe further with some participants. Consequently it was not always easy to fully explore aspects of PPC with patients. This limits the findings to some degree but is illustrative of the wider issues of how complex and sensitive these discussions are for all concerned, within the research setting and between patients, family members and HCPs.

Some interviews with patients and relatives were carried out separately and some jointly. This raises a number of issues, which have been widely debated. Valentine [35] suggests that one of the most valuable aspects of a joint interview is that participants may challenge the other person’s account or provide different perspectives. However, she also identifies the potential to expose underlying tensions between participants – these may be particularly challenging for the researcher to manage when addressing sensitive topics. Others [36] argue that separate interviews are preferable, allowing participants to express their own individual views. However in this argument, there is an implicit suggestion that separate interviews provide ‘truer’ accounts than those accessed by a joint interview. We suggest there is no one definitive approach but a combined approach of joint and separate interviews can provide richer understandings [37] and offer greater potential ‘to explore the complexities and contradictions of the contested realities of shared lives’ [35].

All participants were self-selected, which may suggest some interest and willingness to engage in discussions about in the topics we were exploring. Nevertheless, we found evidence of hesitancy by all parties involved in the initiation of conversations about EOLC preferences; potentially levels of hesitancy or resistance to such conversations may be greater in the wider population The interviews were exploratory and pragmatic in nature with a focus on reported discussion of preferences around EOLC. The findings offers particular insights through triangulation of the follow up interviews with patients/carers and the HCPs involved in the delivery of palliative care. A major limitation of our study is that we were not able to conduct follow up interviews for all the cases. Several factors involved included delays in approvals to approach the selected sites, the involvement of HCPs who had many other priorities on their time and attrition through ill-health and death; these are all factors which impact on research of this nature. The study also had limitations with regard to the cultural mix of participants since all patients recruited to the study were white UK nationals. Research indicates that openness to discussion of preferences for EOLC can differ according to ethnic and cultural background [38] and this is an area which warrants further exploration.

## Conclusion

Despite moves to embed ACP in policy and legal frameworks, its full potential is not being fulfilled. Choosing if, how and when to raise the issue of EOLC preferences, including ACP, is clearly difficult for all concerned. Not all patients in our study expressed a preference to engage in such conversations, which suggests that a uniform approach for HCPs to initiate discussions would not be appropriate. However providing openings to have conversations about EOLC preferences is preferable to not offering the opportunity for patients and family carers to talk about their concerns. Future research is needed to examine the development of interventions to begin the person centred conversations necessary to develop plans to manage EOLC according to patients’ needs and preferences. This work needs to address the benefits of doing so but also management of the risks inherent in the process of having conversations where mortality must be acknowledged.

## Endnotes

^a^This has since been renamed the Preferred *Priorities* for Care but throughout this paper we use PPC to refer to preferred place of care.

^b^http://www.goldstandardsframework.org.uk/

^c^http://www.liv.ac.uk/mcpcil/liverpool-care-pathway/

^d^For example, the National End of Life Care Programme published Advance Care Planning: A Guide for Health and Social Care Staff, August 2008. This was revised again to take account of further developments, and republished in 2011 [25].

^e^A Palliative Care Register is a register of those patients thought to be in need of palliative/supportive care or in the last year or so of life. Setting up a register forms part of the first level of the Gold Standards Framework Programme where adopted by Primary Care Teams in the UK.

^f^Some patients opted to nominate two HCPs in instances where HCPs worked closely together and sometimes made joint visits to patients.

^g^Guidelines from the Royal College of Physicians [24] suggest that professionals should avoid initiating discussions immediately after a move to a care home; discussions are advised to be postponed until once individuals are more settled.

^h^The data were collected immediately prior to the Mental Capacity Act 2005 becoming law in 2007.

^i^All participants were anonymised. Patients were given a number which was also linked to the different study sites. For example Patient 104 is the fourth patient interviewed from Site 1. We have used a generic term HCP for health care professionals interviewed to avoid identification, just indicating the different sites and distinguishing between discussion group interview data (DGP) and follow up interview data (FU). Participants included one GP, several district nurses, community matrons and Macmillan nurses.

^j^In part this may have been because we did not prompt fuller discussions of their preferences. In some instances we also looked for cues of patients, particularly when we had been briefed by health care professionals to take an indirect approach. Some patients quickly changed the subject, several became emotional.

## Competing interest

The Authors declare that there is no competing interest.

## Authors' contributions

KC and JS conceived the project and secured project funding. KC, JS, KA and NM contributed to the design of the study, development of the data collection tools. KA and NM undertook the data collection. All authors contributed to data analysis and helped draft the manuscript. All authors have read and approved the final manuscript.

## Pre-publication history

The pre-publication history for this paper can be accessed here:

http://www.biomedcentral.com/1472-684X/11/15/prepub
